# A Fast Method of Transforming Relaxation Functions Into the Frequency Domain

**DOI:** 10.6028/jres.104.014

**Published:** 1999-04-01

**Authors:** Frederick I. Mopsik

**Affiliations:** National Institute of Standards and Technology, Gaithersburg, MD 20899-0001

**Keywords:** cubic spline, error estimate, Laplace transform, numeric integration, numeric transform, relaxation function, time domain

## Abstract

The limits to the error due to truncation of the numeric integration of the one-sided Laplace transform of a relaxation function in the time domain into its equivalent frequency domain are established. Separate results are given for large and small *ω*. These results show that, for a given *ω*, only a restricted range of time samples is needed to perform the computation to a given accuracy. These results are then combined with a known error estimate for integration by cubic splines to give a good estimate for the number of points needed to perform the computation to a given accuracy. For a given data window between *t*_1_ and *t*_2_, the computation time is shown to be proportional to ln(*t*_1_/*t*_2_).

## 1. Introduction

The transformation of relaxation data recorded in the time-domain into their equivalent in the frequency-domain has been a difficult problem. The data can cover a wide range of times with a correspondingly wide range of frequencies. Both frequency and time are typically measured on logarithmic scales that can cover many decades. What would be desirable is a method for computing the transform to a level of accuracy consistent with the original time data. Also, the time and space requirements should be such that transform can be performed as the data are being acquired so that the frequency domain data can be followed during the course of a long measurement in the time domain.

In a previous paper [1], it was demonstrated that the use of a cubic spline as an integrating tool provided a rapidly convergent method as a function of sampling density for obtaining the Laplace transform of a relaxation function measured as a function of time. The data were assumed to be sampled uniformly on a logarithmic time scale and that the results were displayed on the equivalent logarithmic frequency scale. The method was numerically stable and quite efficient, being order *O*[ln(*t*_2_/*t*_1_)^2^] in computation, where *t*_1_ and t_2_ are the beginning and ending times of the measurement.

Some problems remained in the method. Unlike a conventional FFT type transform, the logarithmic spacing requires the integration of sine and cosine functions that are not harmonically related. Also, the integrals over powers times the sines and cosines must be evaluated very accurately for small arguments where the analytic expressions break down numerically. These conditions require either an extensive computation per point, slowing down the transform, or else the use of a look-up table that can get very large. An examination of the quantities involved in the computation suggested that too much computation was being done.

This paper demonstrates that it is possible to restrict the computation to a predetermined diagonal band transformation matrix such that the computation becomes *O*[ln(*t*_2_/*t*_1_)] and thus far more efficient. As a consequence, the transformation need be defined for at most a normalized frequency decade. This drastically reduces the size of the look-up table so that it can be readily precomputed prior to the main computation, or stored as a table without requiring too much space. Furthermore, the computation becomes almost independent of the size of the data window.

The banding criteria will be developed in terms of a desired tolerance. This will be done separately for both small and large *ωt*. When combined with an estimate for the point density needed to sample a relaxation function with a spline, these results lead to an explicit estimate for the number of points in the band.

It should be noted that these results do not address the problem of continuing the integral outside the data limits. This problem is independent of the computation considered here and has been addressed previously [1], [2]. However, the results presented here do address the question as to how much the missing data outside the measuring window affects the result for any computed frequency.

## 2. Convergence Criteria

Cubic splines have been intensively studied for their convergence properties. These results can be applied directly to the transform of a relaxation function. For the problem considered here, consider a relaxation function *C*(*t*) normalized to vary between 0 and 1, measured in a data window *t*_1_ ≤ *t* ≤ *t*_2_. One wishes to compute the Laplace transform of *C*(*t*_+_) numerically:

$$\begin{array}{c} C^{*}=\int_{t_{1}}^{t_{2}}e^{-st}\frac{dC}{dt}dt,\\ \text{Real}(s)=0_{+}. \end{array}$$ (1)

In the data window this becomes

$$C^{*}=\int_{t_{2}}^{t_{2}}e^{-i\omega t}C^{\prime }dt,$$ (2)

where *C'* is the first derivative of *C* with respect to *t*.

Let *S*(*h*) be the spline passing through *C*(*t*) using a mesh spacing measured by *h*. Then the integral can be written as

$$C^{*}(\omega )=\int e^{-\omega t}S^{\prime }(h)dt+\int {\;e}^{-\omega t}(C^{\prime }-S^{\prime }(h)dt,$$ (3)

where the second term represents the error from using the spline to represent the relaxation function. Since |e^−i*ω*t^| = 1, the second term can be bounded by

$$\left|\int e^{-i\omega t}(C^{\prime }-S^{\prime })dt\right|\leq \int \left|\left(C^{\prime }-S^{\prime }\right)\right|dt\leq \left|C-S\right|.$$ (4)

For a relaxation function that can be represented as a distribution of exponentials, all derivatives exist. Furthermore, if the samples used to determine *S* are uniformly spanned in ln *t*, then the mesh spacing *h* uniformly approaches zero as the sampling density is increased. This leads to the result that

$$\left|\int e^{-i\omega t}(C^{\prime }-S^{\prime })dt\right|\leq \sigma =O(h^{4}),$$ (5)

where *σ* is a tolerance and *h* = ln (*t_j_*/*t_j_*_−1_) [4]. For an exponential, the constant of proportionality for base 10 logorithms can be taken as unity direct computation [1], so that

$$\sigma \leq {\left[1g(t_{j}/t_{j-1})\right]}^{4}.$$ (6)

In general, a relaxation function is not a simple exponential. It can, however, be represented as a distribution of exponentials [5]. For any given exponential, the error term represented in Eq. (6) is a strongly peaked function of *ω* in 1 < *ωt* < 10. Therefore, for a general relaxation function and for a given *ω*, only that part of the distribution with values of *ωt* near the peak in the error term can contribute as much as Eq. (6). Therefore, the error for a relaxation function characterized by a broad distribution of relaxation times must be less than that given by Eq. (6).

## 3. Short Time Limit

Since a cubic spline is a piecewise polynomial, once the spline is fitted to the data, one must evaluate the integrals of the form

$$\begin{array}{l} \int_{a}^{b}x^{n}\sin(x)\;dx,\\ \int_{a}^{b}x^{n}\sin(x)\;dx,\\ n=0,1,2. \end{array}$$ (7)

Only powers to the second degree are needed as the integration is carried out on the derivative of the fitted spline. As noted before [1], the best way to evaluate these integrals for small *x* = *ωt* is to use the Taylor expansion of the integrals. If only the leading term of the Taylor expansions are kept, except for the intgral over cos(*x*), the relative error *R* is given by

$$R<\frac{x^{2}}{2},x<1.$$ (8)

This shows that the explicit integration can be cut off at

$$x=2\sigma^{1/2}.$$ (9)

To this integration, a running sum of the derivative *C'* must be added to the cosine integral which represents the real component of transform.

From Eq. (6), the number of points required per decade is

$$v={\left(\frac{1}{\sigma }\right)}^{1/4}.$$ (10)

This then gives the number of data points *N*_L_ for *x* ≤ 1 as

$$N_{L}=\frac{1}{2\sigma^{1/4}}1g\left(\frac{1}{2\sigma }\right).$$ (11)

For the data corresponding to *x* smaller than the cutoff, one has simply

$$C^{*}(\omega )=C_{j(N_{L})}-C_{1}-\frac{i\omega }{2}\sum_{j=2}^{j(N_{L})}{C^{\prime }}_{j}\left(t_{j}^{2}-t_{j-1}^{2}\right),$$ (12)

where *j*(*N*_L_) is the highest index corresponding to *ωt* < 2*σ*^1/2^. These terms can easily be kept as a running sum.

## 4. Long Time Limit

Whereas the short time data can be kept as a running sum, the long time data beyond a cutoff can be simply disregarded. To show this, consider the long time part of the transform

$$L_{t_{2}}(s)=\frac{A}{\tau }\int_{t_{2}}^{\infty }e^{-st}e^{-t/\tau }dt.$$ (13)

This integral is easily evaluated to give

$$L_{t_{2}}=A\frac{e^{-t_{2}/\tau }e^{i\omega t}}{1+i\omega t}.$$ (14)

Let

$$\begin{array}{c} \chi =\omega t_{2},\\ y=t_{2}/\tau . \end{array}$$ (15)

For a full scale of unity

$$\begin{array}{c} A^{-1}\geq \frac{1}{\tau }\int_{0}^{t_{2}}e^{t/\tau }dt=1-e^{-y},\\ A\leq \frac{1}{1-e^{-y}}. \end{array}$$ (16)

This gives

$$L_{t_{2}}=Ae^{-y}\left[\frac{2}{y^{2}+\chi^{2}}-\frac{i\chi y}{y^{2}+\chi^{2}}\right][\cos\chi -i\;\sin\chi ].$$ (17)

The maximum value is give in the limit of *y* small, *t* >> *t*_1_ to give

$$\begin{array}{c} L_{t_{2}}=\frac{1}{y}\left[\frac{y^{2}}{\chi^{2}}-i\frac{y}{\chi }\right][\cos\chi -i\;\sin\chi ],\\ \approx -\frac{i}{\chi }(\cos\chi -i\;\sin\chi ). \end{array}$$ (18)

Thus, for a tolerance of *σ*, *ωt*_2_ = *l*/*σ*, one obtains for a cutoff from Eq. (18).

$$\omega t_{r}=\frac{1}{\sigma }=\frac{1}{h^{4}}.$$ (19)

This gives the number of data points *N*_H_ for *x* ≥ 1 as

$$N_{H}={\left(\frac{1}{\sigma }\right)}^{1/4}1g\left(\frac{1}{\sigma }\right).$$ (20)

## 5. Final Results

The results given above can be simply combined to give the total width over which the data need to be integrated. This is give from Eqs. (11) and (20) as

$$N={\left(\frac{1}{\sigma }\right)}^{1/4}\left[\frac{3}{2}1g\left(\frac{1}{\sigma }\right)+\frac{1}{2}1g\left(\frac{1}{2}\right)\right].$$ (21)

This expression can be readily evaluated for *σ* = 10^−3^, 10^−4^, and 10^−5^ as 25, 59 and 131 data points respectively.

For a given precision, the frequency spacing should correspond to the sampling density, which is a constant for a given precision. Therefore, the number of frequencies calculated is proportional to ln(*t*_2_/*t*_1_). Also, as the computation is a fixed number of operations for a given frequency, the total computation must be *O*[ln(*t*_2_/*t*_1_)]. This makes the algorithm only slowly dependent on the range of the data and, for wide frequency ranges, very efficient.

In a computation on actual data, since the integrals can be defined in terms of *ωt*, if both *ω* and *t* are equally spaced in log *ω* and log *t*, then only a single row of values need to be stored, since it can be shifted along the data. However, this typically results in awkward frequency values and convenient, rounded values are more desirable. In this case, one only has to use a table large enough to correspond to a normalized decade of frequencies. This is still a great savings in time and storage.

Where the transformation band crosses the edges of the data at *t*_1_ and *t*_2_ mark the points at which behavior outside the measurement window in the time domain affect the desired results in the frequency domain. For the parts of the desired frequency domain where this occurs, one must add the continuation mentioned previously to minimize a possibly large error [2].

In the time-domain instrument in which this algorithm was embedded [2], experimental limitations prevented a true logarithmic spacing for the first few time samples as the sampling rate was not dense enough. This limitation was easily overcome by a subsidiary calculation over the first few data points, until the logarithmic spacing could be properly used, and then picking up the integral table at the appropriate point. While this did introduce some complexity to the program, it had little effect on the running time of the algorithm. For a spline taken at ten points per decade and for the transformation taken for *ωt* from 10^−3^ to 10^5^, the computation was only a few times longer than the spline fitting, which used an over relaxation method that also was nearly *O*[ln(*t*_2_/*t*_1_)] in computation. This band was a little broader than the 3 × 3 10^−2^ to 10^4^ band needed to match the maximum error of 10^4^ to minimize round-off and truncation error for the instrument.

The algorithm has also been compiled into a FORTRAN subroutine^1^ to carry out the numerical transform for a supplied real function. In this case, the time series data window is set by the required frequency window and tolerance. Also, the integration window has been made symmetric about the normalized frequency for clarity in the code so that running sums are not required and only the value of the transformed function at the beginning of the summation is needed for initialization. In this routine, the tolerance is a passed parameter and the routine determines the data window required to perform the transform.

**Fig. 1:**
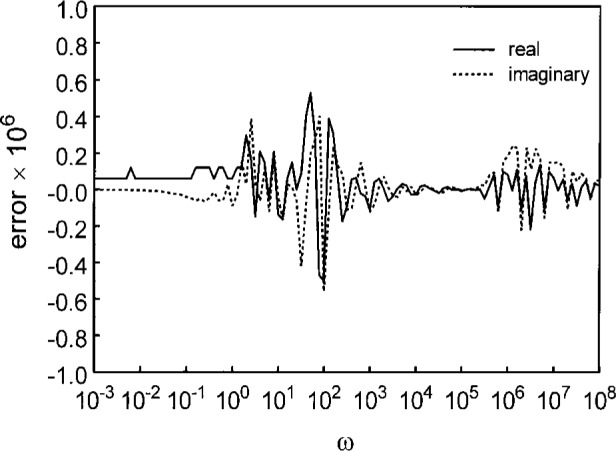
The computational error for a specified tolerance of 10^−6^ for the Cole-Davidson relaxation function with an exponent of 0.5.

For illustration, the time function

$$C(t)=\frac{\gamma (\beta ,t)}{\Gamma (\beta )},$$ (22)

with known transform,

$$C^{*}(\omega )=\frac{1}{{\left(1+i\omega \tau \right)}^{\beta }},$$ (23)

the Cole-Davidson Function [7], where *γ* (*a*, *x*) and G are the incomplete gamma and gamma functions respectively [8], was numerically transformed and compared with the analytic transform. For a requested tolerance of 10^−6^, the maximum computed error was half that. The results are shown in Fig. 1.

## Footnotes

1 The source code is available upon request from the author.
